# Interdependent urban economies: a network analysis of socio-economic relationships between street vendors and formal businesses in Ratnapura, Sri Lanka

**DOI:** 10.3389/fsoc.2026.1882601

**Published:** 2026-10-15

**Authors:** Oshini Kularathne, Lakshika Meetiyagoda, Kokila Sooriyagoda, Nimesh Akalanka

**Affiliations:** 1 Department of Town and Country Planning, Faculty of Architecture, University of Moratuwa, Moratuwa, Sri Lanka; 2 Institute for Geoinformatics, University of Münster, Münster, Germany

**Keywords:** economic network, inequality, public space, social network, street vendors, urban economy

## Abstract

**Introduction:**

The struggle between street vendors and authorities over access to public spaces in urban areas has drawn the attention of scholars for a long time. In the case study of Ratnapura in Sri Lanka, we aim to make visible the social network infrastructure of street vendors with a focus on two major objectives: (1) Exploration of the nature of the relationship between street vendors and formal urban space, and (2) Evaluation of the importance of street vendors for the functions of formal urban spaces.

**Methods:**

The primary data collection methods were systematic observations and semi-structured interviews with 30 participants selected through snowball sampling. The collected data were used to develop activity maps by adapting Håkansson’s Economic Network Model (ENM) and Social Network Analysis (SNA).

**Results:**

The results revealed four types of relationships among the street vendors and formal shop owners—direct, indirect, uni-directional, and bi-directional—based on the three dimensions of actor bonds, activity links, and resource ties. The findings further demonstrate that street vendors interact directly with a wide range of actors and serve as bridges or intermediaries within the urban economic network. Moreover, the results indicate that street vendors do not merely survive in public spaces; they also generate indirect benefits, provide essential services, and support the functioning of other actors within the formal urban system.

**Discussion:**

These findings contribute novel insights to social network analysis research by demonstrating the patterns of socio-economic relationships between street vendors and formal urban actors. Moreover, this research encourages urban planners and policymakers to recognize the contribution of street vendors to urban economic development.

## 1 Introduction

Social networks can play a pivotal role in achieving sustainable production and economy by fostering collective actions, trust, and collaboration at different societal levels (Sonia et al., 2025; Sakamoto, 2024; Zhang et al., 2023). This is especially significant in developing countries, where resources may be limited, and institutional frameworks are often fragile (Magallón and Sáenz, 2026). Understanding social networks to perceive street vendors as having potential for the urban economy is vital, particularly in light of the worldwide promotion of the Sustainable Development Goals of ending poverty (SDG1) (Khan and Shah, 2025), economic prosperity (SDG8) (Thapa et al., 2026), reducing social, political, and economic inequalities (SDG10) (Chowdhary, 2024), and creating inclusive and resilient cities (SDG11) (Addi et al., 2025) by 2030. Achieving these goals requires strategies to increase the economic return of those working within the informal economy, which contributes to 40 percent of the world’s GDP and provides two-thirds of global employment (Bonnet et al., 2019).

The recent systematic review on street vendors by Peimani and Kamalipour (2022) reveals different focuses of existing studies around street vendors: gender (Tamanna et al., 2024; Saxena, 2024), types of street vending (Thinh et al. Sonia et al., 2025), the spatiality of street vending and public space design (Kakani and Tarafdar, 2026; Sandhika et al., 2024), health and wellbeing (Jennifer and Indra, 2025; Sepadi and Nkosi, 2023), policy environment (Wijaya et al., 2021), use of technology (Ramesh et al., 2025; Kumar and Jain, 2025), and links with other forms of informality (Thinh et al., 2025; Kakaza and Naude, 2025). Furthermore, recent studies have examined the role of the social network of street vendors (Anja and Zhang, 2023). Gogoi and Thakur (2023) examined migrant street vendors; Navarrete-Hernández et al. (2023) explored the social network as a street vendor’s poverty alleviation strategy; and Zhang et al. (2023) investigates the opportunities and challenges confronted by social network interventions in the vendor economy. However, a study investigating street vendors’ social networks to understand their relationship with the formal economic structure is limitedly explored. The literature indicates that social networks are key to the survival of street vendors and their continued use of space (Forkuor et al., 2017; Milgram, 2011; Crossa, 2009). It also identifies the relationship between street vendors and city users (Wanigasinghe and Meetiyagoda, 2026), but few studies explain the patterns of economic activity-based social relationships. Understanding the patterns of how social networks emerge, grow, are sustained, and relate to formal and informal urban economic systems can help scholars grasp the depth of these relationships and assist planners in devising effective and equitable urban planning strategies. In this context, this research examines the role of street vendors within the formal economic space. Accordingly, based on data collected from 30 participants who were selected using the snowball sampling method, the social network actors were identified. Thereafter, Hakansson’s Economic Network Model (ENM) and Social Network Analysis (SNA) were adapted to understand, visualize, and discuss the nature of the relationship between street vendors and formal urban actors and their impact on the existence of the formal urban economic structure and the space.

The findings of this study on the examination of hidden social networks within the formal urban economic system have significant theoretical and practical importance. From a theoretical perspective, this study enhances the understanding of urban economic networks by emphasizing the intricate social relationships of street vendors, which are frequently disregarded in urban planning literature. The study examines the interactions between street vendors and the formal economy, addressing a significant need in urban studies, which is crucial for urban planners and policymakers. This study highlights the important contribution of street vendors to urban economies and proposes that acknowledging and incorporating these informal networks can aid in formulating policies that foster inclusive and robust urban economic development. The study supports the idea of including street vendors in formal economic systems, which can lead to regulations that improve their role in promoting inclusive and sustainable urban development.

Thus, we pursue this examination in the remaining sections of the article as follows. We begin with a brief overview of Church Road, Ratnapura (the study context) and a discussion of the data and methods employed, followed by a discussion of the methods of analysis based on Hakansson’s Economic Network Model (ENM) and Social Network Analysis (SNA) to examine the role of street vendors within the formal economic space. We then present the results of our analysis under two phases: (1) Exploration of the nature of the relationship between street vendors and formal urban space and (2) Evaluation of the importance of street vendors for the functions of formal urban spaces, explaining complex relationships within the urban economy and underscoring the substantial influence of street vendors. We conclude by reflecting on these results within the framework of urban economic policy and the impact of the informal sector on development. The paper emphasizes the need to incorporate street vendors into formal economic structures and advocates for further research to strengthen sustainable urban development.

## 2 Literature review

### 2.1 Informality and the informal economy in formal urban spaces

Scholars argue that people informally shape “formally” constituted urban built environments to appropriate spaces for their activities, symbolize “daily life” (Lefebvre, 2014), and provide opportunities for ordinary people (Perera, 2015).

As scholars suggest, the formal and informal should not be understood as opposite and separate domains (Koster and Nuijten, 2016; Bunnell and Harris, 2012; McFarlane, 2012). As Dovey (2012, p. 363) points out, ‘the informal/formal conception is both fundamental and non-dichotomous—it is a single twofold concept rather than two concepts in opposition’. In all major and minor urban areas of Sri Lanka, informal spaces for vending, trading, parking, and meeting are common within planned spaces (Pathirana and Munasinghe, 2021). The informal economy underpins urban informality. Williams and Round (2009) define the informal economy as permissible goods and services that are unregistered or hidden from the government for taxation and benefits. Within this context, street vending is a major form of informal employment in cities (Basu and Nagendra, 2020; Asiedu and Agyei-Mensah, 2008; de Pádua Carrieri and Murta, 2011; Crossa, 2014).

As Bhowmik (2007) stated, activities in the informal sector can be divided into two categories: self-employment and casual labour, with street vending representing a substantial portion of the self-employed category. Thus, street vending is an ancient and significant occupation within the informal sector (Bromley, 2000) and is prevalent in almost all developing and developed countries (Bhowmik, 2010; Karunaratna and Tjandra, 2021).

Due to the high mobility of street vendors and the nature of their operations, it is difficult to determine how many people make a living through vending (Karunaratna and Tjandra, 2021; Roever and Skinner, 2016). Some are part-time sellers, while others run their businesses as family enterprises, with family members joining intermittently throughout the day, one in the morning and another in the evening (Karunaratna and Tjandra, 2021). The commodities sold, hours worked, locations, and business acumen of street vendors make it difficult to quantify their income (Devlin, 2011). According to Yatmo (2008), street vendors’ ability to move, install, and dismantle their trade stalls suggests that they can be considered semi-formal or informal vendors operating with temporary urban components.

When considering the behavior of street vendors in formal urban spaces, their concentration or dispersion is not uniform throughout the city (Panwar and Garg, 2015). It is essential to understand why they select particular places or locations for their businesses. They tend to concentrate in specific locations that often experience high levels of pedestrian and vehicular congestion (Bromley, 2000). Sharma (2007) explains how street vendors focus on the usual paths that users travel daily on their way to work or other businesses. According to Mitullah (2003), selecting a location with substantial pedestrian traffic and potential customers is crucial for the survival of street vendors. In addition, consumers prefer shopping and engaging in social interaction in the most active public spaces (Sarkar, 2016).

Street vendors remain vulnerable to government persecution (Recio and Gomez, 2013) because they may block sidewalks, disrupt traffic, and create congestion (Bhowmik and Saha, 2012). Due to the nature of their occupation, street vendors encounter numerous obstacles and difficulties (Karunaratna and Tjandra, 2021). In addition, their use of public space may contribute to their perceived illegality, as they do not own or lease the spaces used for vending and are therefore often viewed as invaders or trespassers (Brown, 2006; Yatmo, 2008). The activities of street vendors are often perceived as unsupervised and unorganized, which has caused several issues, particularly for pedestrians (Karunaratna and Tjandra, 2021; Widjajanti et al., 2019). Therefore, local governments have introduced various policies to control and regulate street vending, such as registering street vendors and permitting them to vend, limiting street vending to designated locations, and evicting vendors from unauthorized locations (Karunaratna and Tjandra, 2021; Yatmo, 2008).

In this context, eliminating street vendors from urban areas has become “a valiant initiative to organize the environment” from the perspective of government (Douglas, 1966) and is viewed as an indicator of urban development (Bromley, 2000; Koster and Nuijten, 2016). Street vendors are now a vulnerable sector that frequently loses the space battle in contemporary urban environments (Yatmo, 2008). However, debates regarding street vendors in urban settings have developed along two opposing paths. Accordingly, many perceive them as a nuisance in cities, while other factors encourage street vending in urban environments (Bromley, 2000). These opposing views have contributed to ongoing debates about the role of street vendors in cities (Yatmo, 2008).

Taken together, the literature demonstrates that street vending is deeply embedded in the social, spatial, and economic life of cities, and that relationships and social networks are important for vendors’ survival and continued access to urban space. However, existing research has predominantly examined these networks from the perspectives of vendors’ livelihoods, social support, poverty alleviation, or relationships with customers and other informal actors, while the socio-economic connections between street vendors and formal economic actors within shared urban spaces remain comparatively underexplored. In particular, limited attention has been given to how social relationships shape economic activities between street vendors and formal vendors, how these relationships are structured through actor bonds, activity links, and resource ties, and how such interactions contribute to the functioning of formal urban spaces. This represents an important gap because the formal and informal economies are not separate systems but are often interdependent and spatially interconnected. Addressing this gap requires an approach that can simultaneously reveal the structure of social networks and the economic relationships embedded within them. This study therefore examines the socio-economic network relationships between street vendors and formal urban actors in Ratnapura, Sri Lanka, by adapting Håkansson’s Economic Network Model and Social Network Analysis to identify the patterns of interaction, interdependence, and resource exchange that connect informal vending with the formal urban economic system.

### 2.2 Social Network Theory (SNT)

Several theoretical perspectives have been used to study social relations within urban informality, including social capital theory (Portes, 1998), the embeddedness of economic action in social relations (Granovetter, 1985), Actor-Network Theory (Latour, 2005), and theories of urban informality as a mode of urbanization or a form of quiet, everyday politics (Roy, 2005; Bayat, 2000).

Social Network Analysis (SNA) operationalizes these aspects of social networks by examining the relationships and connections among actors. A node, or ego, is the basic element of a social network. Each actor is represented as a ‘node’, while the connection or linkage between nodes is referred to as an ‘edge’ or ‘tie’ (Carrington, 2014) (Figure 1).

**Figure 1:**
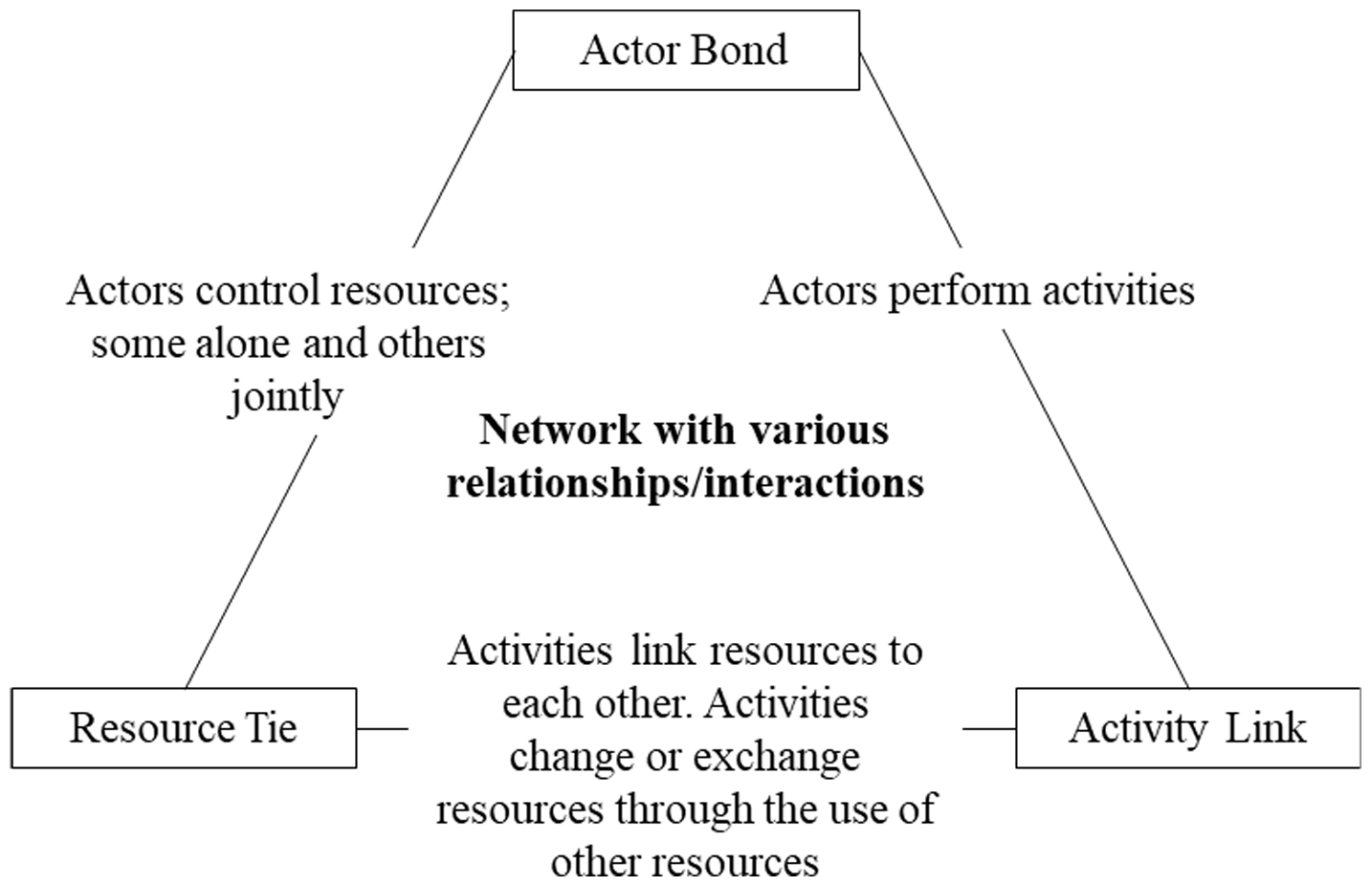
Interplay of three layers in ENM. Source: Håkansson and Snehota, 1995.

Given the two related terms used in this study, a distinction is made between Social Network Theory (SNT) and Social Network Analysis (SNA). SNT provides the theoretical framework through which the relationships identified in this study are interpreted. As discussed by Adam and Kriesi (2007), social network perspectives explain why relationships between actors matter, particularly how ties, exchanges, and structural positions within a specific group or space influence information transfer, collaboration, and patterns of interaction. This perspective also seeks to understand the social ties, exchanges, and flows that structure the settings in which vendors operate and shape their relationships with formal urban spaces (Zook, 2017).

SNA, by contrast, is the analytical approach used to operationalize these relationships. It provides a set of procedures for representing observed relationships as a formal network consisting of nodes and edges and for examining the structural characteristics of that network through measures such as centrality (Marsden, 1990; Scott, 2012; Wasserman and Faust, 1994; Carrington et al., 2005). In SNA, a node represents an actor or entity within the network, while an edge or tie represents a relationship or connection between two nodes (Carrington et al., 2005). The term ‘ego’ refers specifically to a focal actor in an ego-centred network and is therefore not necessarily synonymous with a node.

Centrality measures are used in SNA to identify the relative structural importance or position of actors within a network (Newman, 2010). Rather than simply referring to the impact of removing an actor from the network, centrality captures different dimensions of an actor’s position and connectivity within the network. In this study, three measures of centrality were examined: degree centrality (DC), betweenness centrality (BC), and closeness centrality (CC).

*Degree Centrality (DC)*: Degree centrality refers to the number of direct ties that a node has with other nodes in the network (Zook, 2017). In simple terms, it indicates how many other actors are directly connected to a particular actor. Higher degree centrality indicates a greater number of direct connections and can therefore be used to identify highly connected or focal actors within the network (Ratnayake et al., 2016).

*Betweenness Centrality (BC)*: Betweenness centrality identifies actors that occupy intermediary or bridging positions within a network (Zook, 2017). It measures the extent to which a node lies on the shortest paths between other pairs of nodes. Actors with high betweenness centrality may therefore function as bridges or intermediaries, potentially influencing the movement of information, resources, or interactions between otherwise less-connected actors.

*Closeness Centrality (CC)*: Closeness centrality measures how close a node is to all other nodes in the network. It is generally calculated based on the shortest-path distances between a node and all other reachable nodes (Zook, 2017). Actors with high closeness centrality are positioned relatively close to other actors and can therefore potentially reach or interact with other parts of the network efficiently.

### 2.3 Hakansson’s Economic Network Model (ENM)

Hakansson et al. (1982) developed and adapted an actor network concept called the ‘Economic Network Model’. It was created to examine the nature of interdependencies, interconnections, and relationships of business ties. In his theory, Hakansson emphasizes the importance of mutual direction and commitment in the relationship between two actors/business organizations/companies. The relationship among them generates activities and resources that are interdependent (Damayanti, 2010).

Hakansson also concluded that every connection has unique characteristics and nature. These characteristics are affected by three distinct levels of the relationship’s structure (Damayanti, 2010).

- Layer 01 – Activity Link ➔ Technical, organizational, and other operations that might be linked in a relationship.
- Layer 02 – Resource Tie ➔ Resource ties that link diverse aspects of a corporate organization with one another. These shared resources include both tangible (materials, technology) and intangible (knowledge) components.
- Layer 03– Actor Bond ➔ A link between actors impacts how they perceive, evaluate, and treat one another (Håkansson and Snehota, 1995; Oerlemans et al., 1998; Fayezi and Zomorrodi, 2015).

All three layers configure connections as depicted in Figure 1.

**Figure 2:**
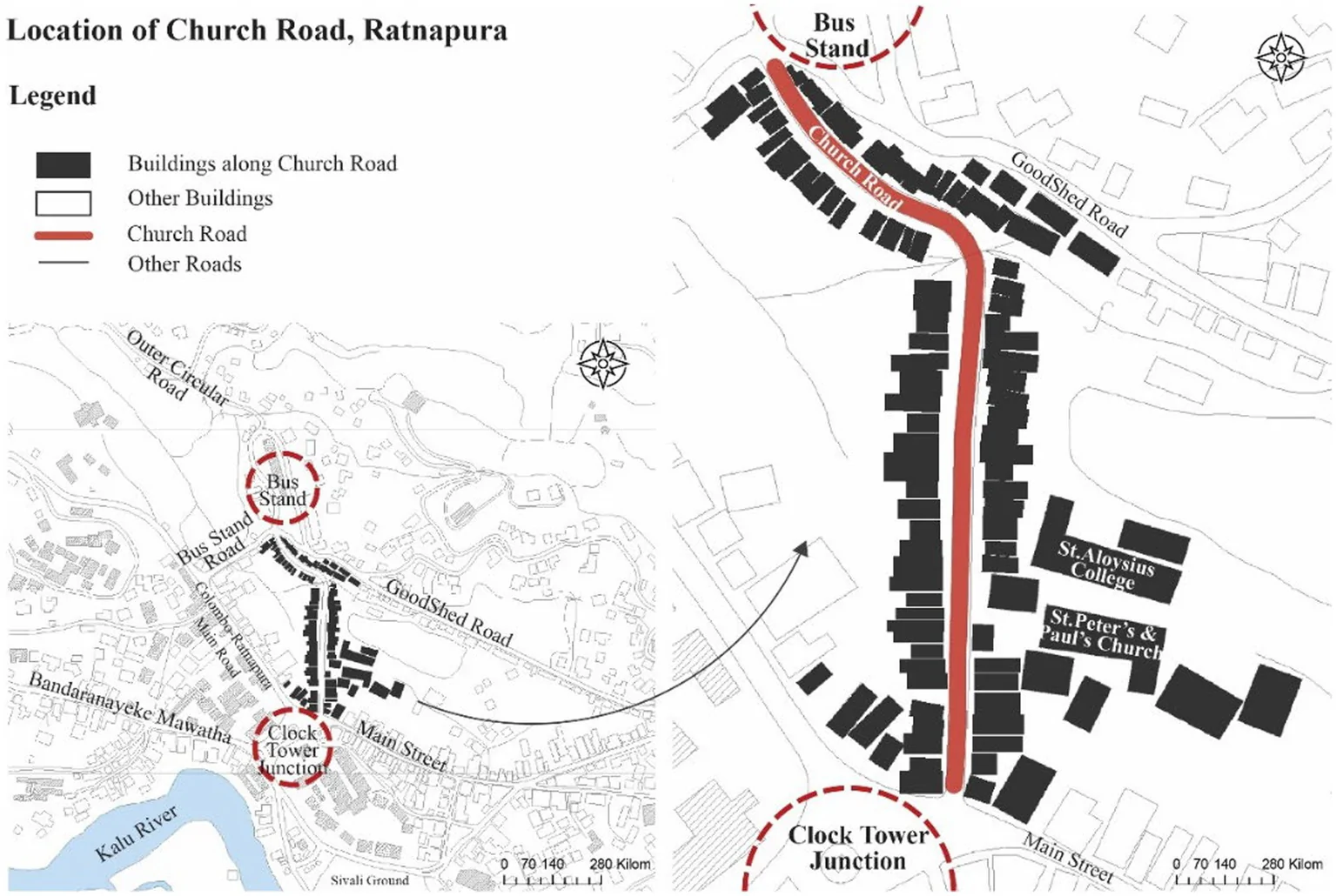
Location of Church Road. Source: prepared by the author.

Its purpose was to investigate the characteristics of interdependencies, interconnections, and business relationship ties. Hakansson’s theory places significant emphasis on the reciprocal influence and dedication that characterize the relationship between two entities, be they organizations or businesses. An interdependent flow of resources and activities results from their relationship (Damayanti, 2010). A study conducted by Sabine et al. (2014) illustrates how business relationships can be explained using the Economic Network Model and how that relationship exists if there is a mutually oriented interaction between two reciprocally committed parties over time. Furthermore, Gadde (2023) has applied this Economic Network Model to understand the actor networks in the car industries in the USA and Europe, identifying that relationships create interdependencies over time in terms of technology, knowledge, social relations, administrative routines, and legal ties.

## 3 Materials and methods

### 3.1 Study area—Church Road, Ratnapura

The selected study area, Church Road, is in the center of Ratnapura Town, the provincial capital of Sabaragamuwa Province, Sri Lanka (Figure 2).

**Figure 3:**
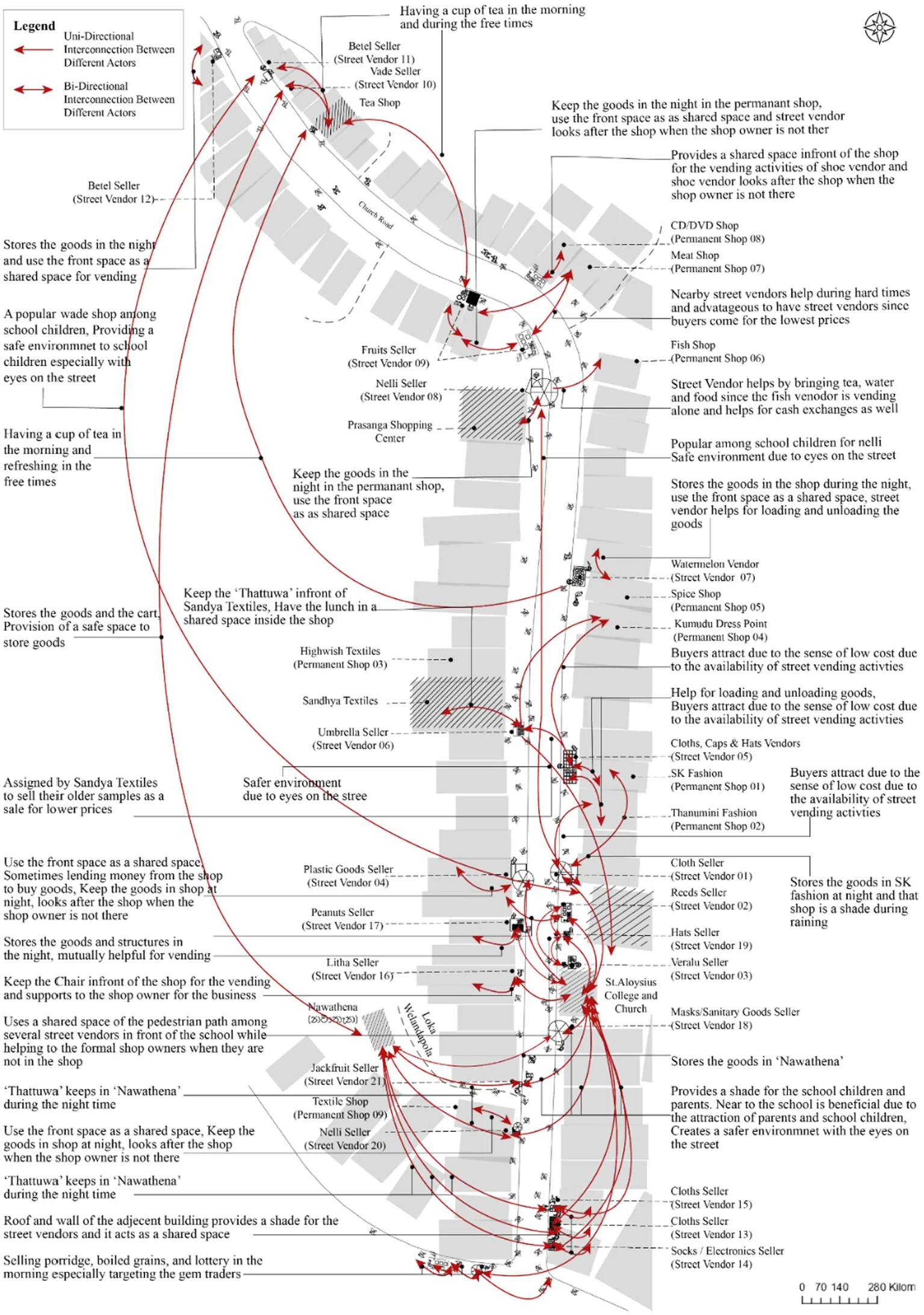
Activity map. Source: prepared by the author.

Ratnapura is a regional transport hub that connects the Eastern, Southern, and Central Provinces. As of 2021, Ratnapura, being the provincial capital and a major transit hub, attracts over 100,000 daily commuters from the region (Urban Development Authority-Ratnapura, 2019). The well-established gem industry and trade have shaped the economic geography of Ratnapura Town. The centralized administrative functions, education, and newly improved health facilities have also helped develop other supporting economic activities, especially in accessible locations and routes. Accordingly, Church Road is a vital part of the town core of Ratnapura, as it serves as an important corridor for commercial and trading activities, the gem industry, and educational pursuits. It also makes a substantial contribution to the city’s economy (Urban Development Authority - Ratnapura, 2019).

Church Road in Ratnapura is 310 metres in extent. It is formed by linking the Colombo-Ratnapura Main Road from the Clock Tower Junction and the Good Shed Road from the bus stand premises. Furthermore, a significant number of pedestrians utilize Church Road as an alternate route or bypass to reach Good Shed Road, the premises of the bus stand, Colombo-Ratnapura Main Road, Main Street, and Clock Tower Junction.

**Figure 4:**
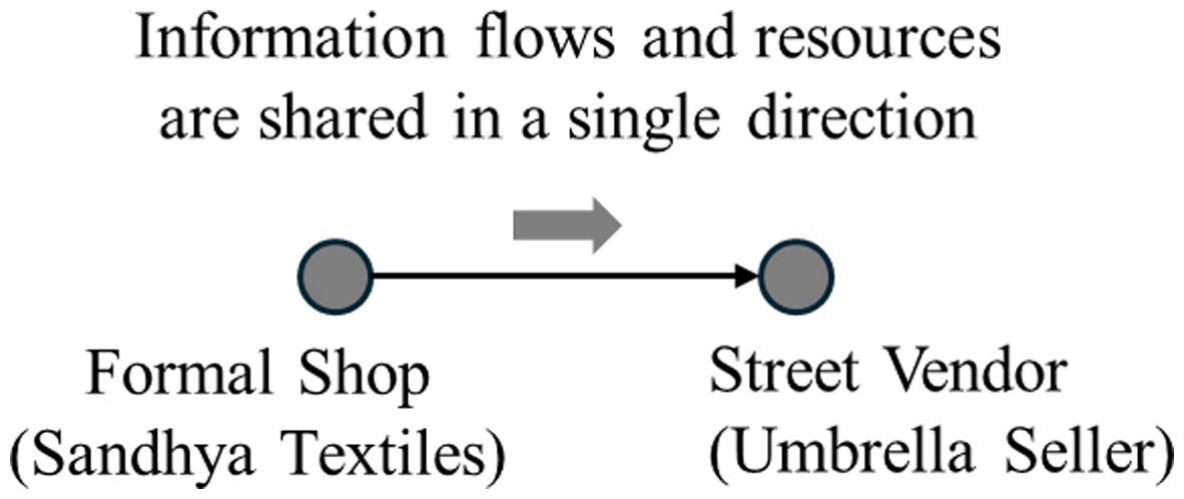
Direct and uncomplicated interaction between two individuals or entities. Source: prepared by the author.

**Figure 5:**
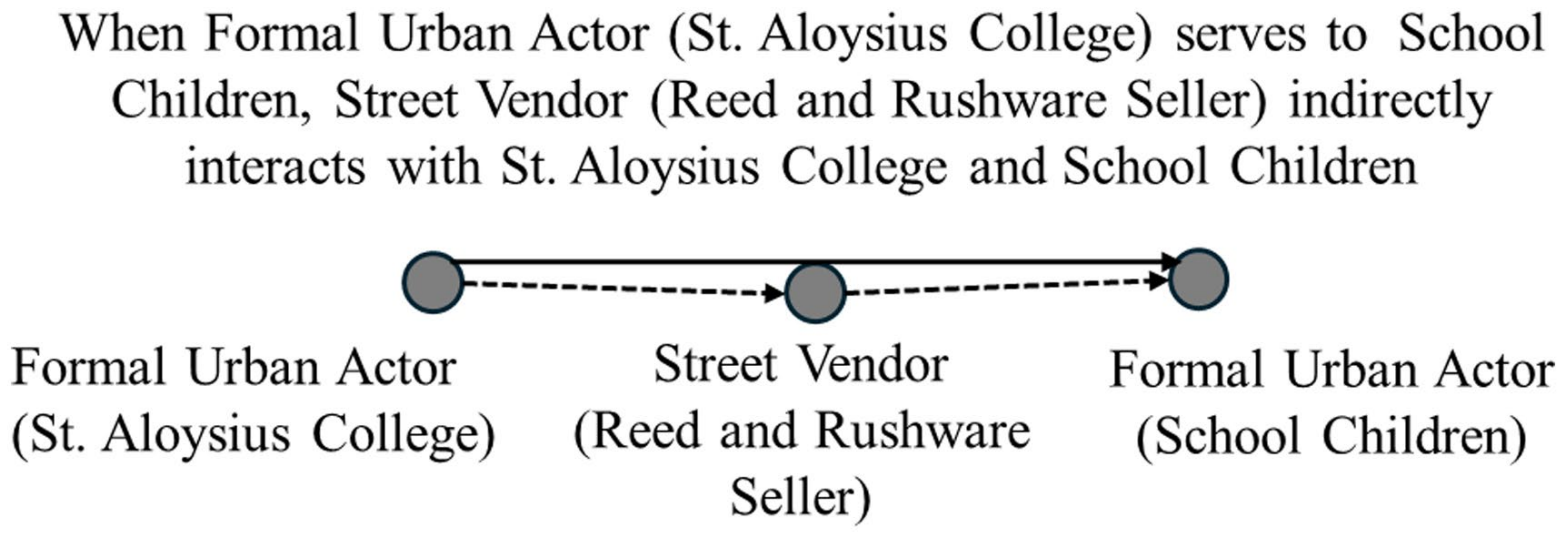
Indirect relationship between two individuals or parties established for another purpose, activity, or interaction. Source: prepared by the author.

As a result, a considerable proportion (approximately 70–80%) of street vendors are setting up businesses on Church Road informally due to the road’s advantageous location and greater economic potential. Moreover, Church Road in Ratnapura has been recognized as a prominent street vending zone in the city centre (Urban Development Authority - Ratnapura, 2019). In comparison to other commercial areas in Ratnapura City, this area offers a wide range of products at a significantly reduced cost, satisfying the diverse needs of consumers.

Church Road in Ratnapura is renowned for its street vending, but it also comprises a multitude of permanent commercial structures, including St. Peter’s Church, St. Paul’s Church, and St. Aloysius College, among other formal urban spaces. Furthermore, the social and structural configurations of Church Road exhibit a dynamic quality as the day progresses. In addition, this thoroughfare is entirely pedestrianized throughout the festival seasons due to the greater concentration of street vending activities and the surge in pedestrian traffic. Accordingly, Church Road was selected as the focus of this study due to its importance as a pivotal, economically dynamic thoroughfare in Ratnapura. This corridor is crucial for comprehending the interplay among urban infrastructure, the dynamics of street vending, and economic operations in a significant provincial capital and transportation node.

### 3.2 Research design

This study is guided by two research objectives: (1) to explore the nature of the relationships between street vendors and formal urban actors, and (2) to evaluate the importance of street vendors for the functioning of formal urban space. These objectives shaped the choice of data collection methods. Because much of the interaction between street vendors and formal urban actors is informal, undocumented, and embedded in ongoing personal relationships rather than in official records, addressing Objective 1 required methods capable of capturing context-specific behavior and interaction *in situ*. For this reason, on-site documentation, systematic observation, and semi-structured interviews were selected as the primary data collection methods, as these are well suited to studies requiring close observation of behavior and immersion in a specific community (Punch, 2005; Caulfield, 2022). Addressing Objective 2, in turn, required a method capable of systematically structuring and quantifying these qualitative relational patterns in order to assess their comparative significance within the urban economic system. Håkansson’s Economic Network Model and Social Network Analysis were therefore adopted as complementary analytical tools, allowing the qualitative relational data gathered through observation and interviews to be coded, mapped, and evaluated for structural importance.

With these fundamental research methods, the study was designed predominantly as qualitative, with only a rudimentary quantitative approach, in order to understand the relationship between street vendors and other urban actors, as well as the formal urban space. Williams and Shepherd (2017) suggest that employing such an approach to understand networks or relationships enhances the ability to investigate broader networks, providing greater access to diverse social actors, reducing informant bias, and increasing the capability for longitudinal designs.

However, this study was conducted mainly using various qualitative data collection methods, including on-site documentation, systematic observations, and semi-structured interviews. The utilization of these qualitative methods allowed for a deeper understanding of how individuals perceive their environments (Punch, 2005). Furthermore, Caulfield (2022) emphasized that these qualitative data collection methods are optimal for studies that require close observation of people’s behavior and interactions. Additionally, this study utilized multiple qualitative data collection methods, as it has been suggested that employing multiple methods to investigate a phenomenon yields more reliable and persuasive results compared to single-method studies (Davis et al., 2011; Heath et al., 2018).

First, preliminary observations were conducted to understand the context. Karunaratna and Tjandra (2021) imply that observations can be an effective qualitative data collection tool to detect the nature of the behavior of street vendors in a study conducted to analyse the key attributes of street vending in the context of Sri Lanka. Accordingly, in this study, around 70 to 80 street vendors engaging in their vending activities along Church Road were identified. The on-site documentation was done simultaneously to identify street vendors and their locations, observe their ties and relationships with formal urban spaces, how they engage with them, their activity patterns, and external physical aspects. Observations were carried out on both weekdays and weekends during three time periods covering the morning, afternoon, and evening by the author (Table 1).

**Table 1:** Identified activities and relationships between street vendors and formal urban space during the three observation periods of the day.

Observed time period	Identified activities/relationships	Actors responsible for the identified relationship
Morning (6.30 a.m.–12.00 p.m.)	Arrival of the vendors to Church Road around 7.00 a.m.	-
	The vendors (both street vendors and formal shop owners) who come early to Church Road have some small conversations with each other about their last-day businesses and share their thoughts about different topics such as politics, etc.	Street vendors Formal shop owners/vendors
	Commencing their operations by erecting their *‘Thattuwa’* (the structure that vendors use to keep the goods and their selling items for selling) and stalls in the open space in front of the formal permanent shops or on the pedestrian path, while it acts as a shared space between street vendors and formal urban spaces.	
	Some street vendors are assigned by the formal shops to sell their items as a sale in front of their shops.	
	Concentration of gem traders around the Clock Tower Junction (one end of Church Road) street vendors sell porridge, boiled grains, and lotteries targeting gem traders.	Street vendors Gem traders
	There is a tea shop on Church Road which is popular among street vendors and other formal vendors, and they visit this tea shop and have a cup of tea before starting their vending activities in the morning or during their free time.	Street vendors Formal shop owners/vendors Tea shop
	School children, their parents, and people who go to work use this road to access their school and workplaces, and vending activities are higher from 9 a.m. - 12 noon.	-
Afternoon (12.00 p.m.–3.00 p.m.)	During the 1.30 p.m.-3.00 p.m. time, most buyers are the parents who come to pick up their children from St. Aloysius College.	-
	Street vendors sell complementary goods such as stickers, sweets, and stationary targeting school children around St. Aloysius College at a very low price.	Street vendors School children
	Street vending structures provide shade for parents who are waiting for their kids from school, as well as for the school children who are waiting for their parents or school vans.	Street vendors School children Parents
	Nearby permanent shops and formal urban structures provide shade for many street vendors during heavy rains.	Street vendors Formal shop owners/vendors
	Some street vendors conduct their vending activities under the shadows of tall buildings, in front of some shops, under the shades of trees, etc.	Street vendors Formal shop owners/vendors Surrounding environment
	Street vendors navigate the buyers to the formal vending premises/ shops that buy the goods from street vendors.	Street vendors Formal shop owners/vendors
	Street vendors support the formal shops on Church Road with loading and unloading the goods, for the vending activities when the owners are not in the shop, etc.	
	Street vendors pay Rs.50 per day to Ratnapura Municipal Council (RMC) for selling their items along Church Road	Street Vendors Ratnapura Municipal Council (RMC)
Evening (3.00 p.m.–6.30 p.m.)	The vending activities are higher from 3.00 p.m. – 6.00 p.m.	-
	Higher use of Church Road as an alternative route to access the Bus Stand by the pass-by pedestrians from 4.00 p.m.—6.00 p.m., fulfilling their daily needs while going back to their homes.	Street vendors Formal shop owners/vendors Pass-by pedestrians
	Street vendors store their goods in a variety of secure locations overnight, such as nearby shops and designated places like ‘Nawathena’ or along the road.	Street Vendors Formal Shop Owners/vendors ‘Nawathena’ Road

Source: prepared by the author.

Then the Semi-Structured Interviews were conducted, focusing on street vendors and surrounding urban actors. According to statistical theories, for qualitative data collection methods, a sample size of 5 to 50 participants is adequate (Dworkin, 2012; Mason, 2010; Moser and Korstjens, 2018) because a small sample allows the researcher to focus on in-depth understanding in a particular social and cultural context, which is generally not possible when studying larger samples (Subedi, 2021). Accordingly, 30 participants were selected as the sample, including 21 street vendors and 9 shop owners. Data collection continued until saturation was observed in the relational patterns emerging from the interviews: the final two to three interviews conducted with each actor type (e.g., the last shop owners interviewed) confirmed the actor bond, resource tie, and activity link patterns already identified in earlier interviews, rather than revealing new categories of relationships, indicating that the sample of 30 participants was sufficient to capture the range of relationships relevant to this study. This sample size is further consistent with recent qualitative studies conducted in comparably sized Sri Lankan urban settings; for instance, Wanigasinghe and Meetiyagoda (2026) similarly interviewed 30 participants in a single-case mixed-method study of street vendor–city user relationships in Ragama town, a medium-sized town in the Colombo metropolitan region, lending further support to the adequacy of this sample size for the present study’s context and scope.

Based on the research objectives and the purpose of the study, the Snowball Sampling method was used to derive the sample, as the participants/informants of this study could not be predetermined. Thus, the interconnected actors of the network were identified through the previously known informant. Therefore, in this method, respondents (participants) who have previously been in contact with the researcher use their social networks to suggest or recommend other persons who might be able to take part in or contribute to the study (Mack, 2005). Furthermore, this snowball sampling method can detect the network between the hidden actors within the street vending culture because it is often used to find and recruit “hidden populations” that are not easily accessible or detectable through other sampling strategies (Naderifar et al., 2017).

During the semi-structured interviews, a specific code was assigned to each street vendor and shop owner/formal vendor, which was applied only to the 30 informants focused on during the semi-structured interviews. Accordingly, ‘SV’ refers to Street Vendors, and ‘SO’ refers to shop owners/formal vendors. The themes covered in the semi-structured interviews included the duration of their vending activities, the sources and methods of obtaining goods, how they store or keep their goods at night, their interactions and relationships with nearby shops and vendors, and the benefits they gain from these interactions.

The data analysis was conducted in two major phases in this study: Phase 1—Exploration of the nature of the relationship between street vendors and formal urban space, and Phase 2— Evaluation of the importance of street vendors for the functions of formal urban spaces. The first phase of the analysis was based on qualitative methods. An activity map was prepared based on the collected data, including the locations of the actors on Church Road, their various interconnections, and linkages. Building upon that, the patterns of relationships were identified as: (1) direct relationships, (2) indirect relationships, (3) uni-directional relationships, and (4) bi-directional relationships, which helped to derive the nature of the relationship. Based on this, the reasons for each linkage between different actors were sketched and interpreted as cross-sections to qualitatively analyse and understand the nature of the relationship between the informal street vendors and the formal urban spaces based on Hakansson’s Economic Network Model (ENM). This study adopts Hakansson’s Economic Network Model (ENM) for two main reasons. First, unlike social capital theory and embeddedness, which conceptualize social relations broadly in terms of trust, norms, reciprocity, or the general social context of economic action, ENM decomposes a relationship into three distinct, empirically codable layers: Activity link, resource tie, and actor bond, which separately capture the operational/technical, resource-based, and relational/attitudinal dimensions of an economic tie. This layered structure offers a more operational coding scheme for translating the qualitative interview and observation data into discrete analytic categories, which is central to the aims of this study. Second, unlike Actor-Network Theory, which treats the tracing of associations between human and non-human actors as an open-ended, descriptive exercise, and unlike broader theories of urban informality, which are primarily concerned with informality as a socio-political condition or mode of urbanization rather than with the internal structure of specific relationships, ENM was originally developed to examine inter-organizational and inter-actor relationships within economic systems (Håkansson and Snehota, 1995), which aligns directly with this study’s aim of mapping and quantifying the economic-functional relationships between street vendors and formal urban actors. Furthermore, because the coded ENM layers could be directly translated into a node-and-edge dataset, ENM provides a natural bridge to Social Network Analysis, allowing the qualitative relational patterns identified in Phase 1 to be quantitatively structured and analysed in Phase 2—a translation that is less straightforward when relying solely on social capital theory, embeddedness, or urban informality frameworks. ENM has similarly been applied to explain inter-actor relationships within other economic geography contexts (Sabine et al., 2014; Gadde, 2023), providing further precedent for adapting this framework to the urban economic context studied here.

Håkansson and Snehota (1995) further deduced that each connection possesses distinct qualities in this Economic Network Model. By observing how these attributes are influenced by three discrete tiers of the relationship’s architecture (Damayanti, 2010), one can discern the true nature of the relationships that exist among individuals.

- Layer 01 – Activity Link - Technical, organizational, and other operations that might be linked in a relationship.
- Layer 02 – Resource Tie - Resource ties that link diverse aspects of a corporate organization with one another. These are the shared resources, including both tangible (materials, technology) and intangible (knowledge).
- Layer 03 – Actor Bond - A link between actors impacts how they appear, appraise, and treat one another (Håkansson and Snehota, 1995; Oerlemans et al., 1998; Fayezi and Zomorrodi, 2015).

To operationalize the ENM, the reasons for each relationship pattern identified through the transcribed interviews and the on-site observation notes underlying the activity map were coded deductively against these three layers for every identified actor pair (e.g., street vendor–formal shop, street vendor–pedestrian, street vendor–institution). Coding followed the three interview/observation themes reported above: references to concrete, recurring operational tasks performed jointly or in sequence by the two actors (e.g., loading and unloading goods, jointly using a queue or selling spot) were coded as an activity link; references to the use, provision, or sharing of a tangible or intangible resource between the two actors (e.g., pavement or shop space, storage, information) were coded as a resource tie; and references to the relational quality between the actors, including mutual recognition, tolerance, or informal obligation expressed by the informant when describing the other actor (e.g., being allowed to stay, being looked out for), were coded as an actor bond. Each of the three layers could be coded as present or absent for a given actor pair, and a pair was retained as an analytic unit if at least one layer was identified. The coded actor pairs with different relationship patterns were then synthesized into the cross-sectional relationship sketches presented in Section 3.1 and summarized in Tables 2, 3, which report, for each example, the specific actor bond, resource tie, and activity link identified from the underlying interview and observation data.

**Figure 6:**
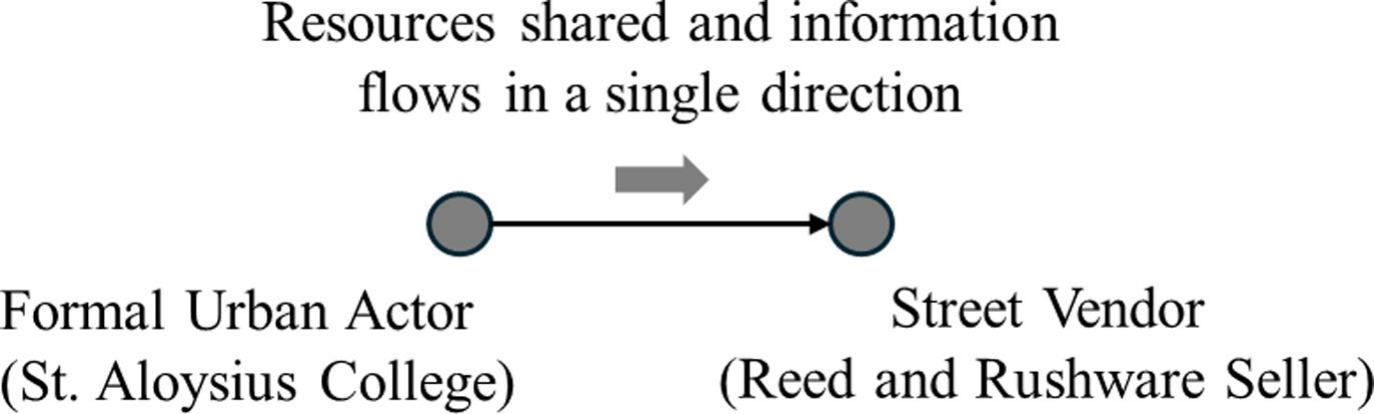
An asymmetrical relationship characterized by one side providing or exerting influence over the other without receiving any reciprocation or feedback. Source: prepared by the author.

**Figure 7:**
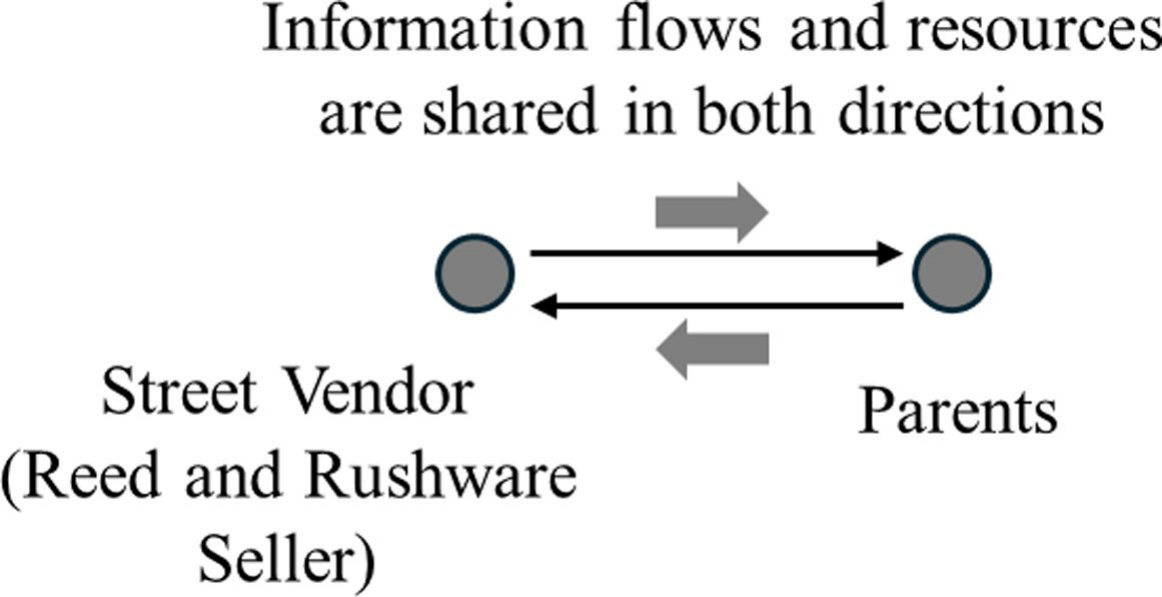
A reciprocal relationship, with an interdependent and mutually beneficial nature, is formed when both parties engage in mutual interaction and exert influence on each other. Source: prepared by the author.

**Figure 8:**
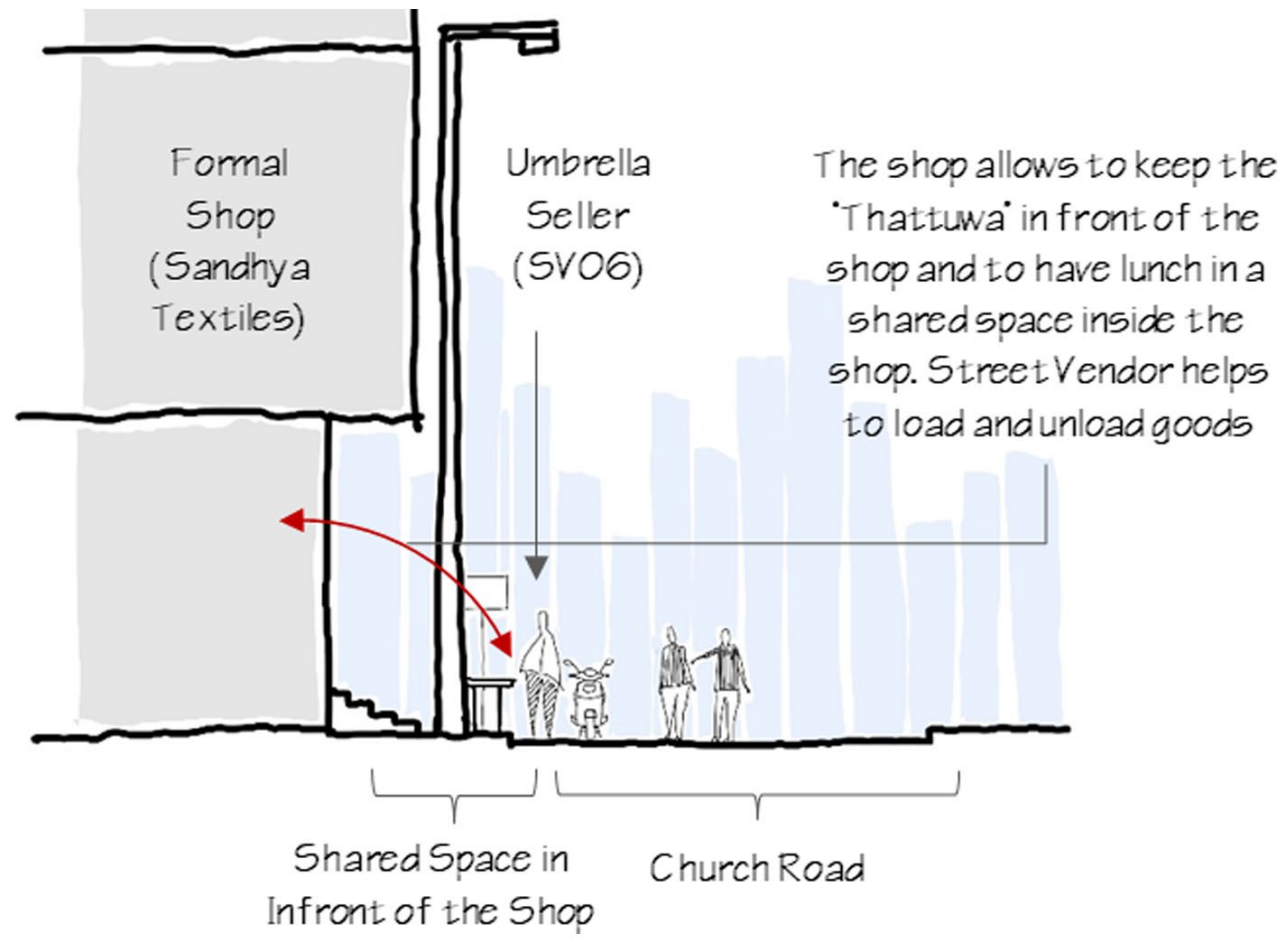
Example 01. Source: prepared by the author.

**Figure 9:**
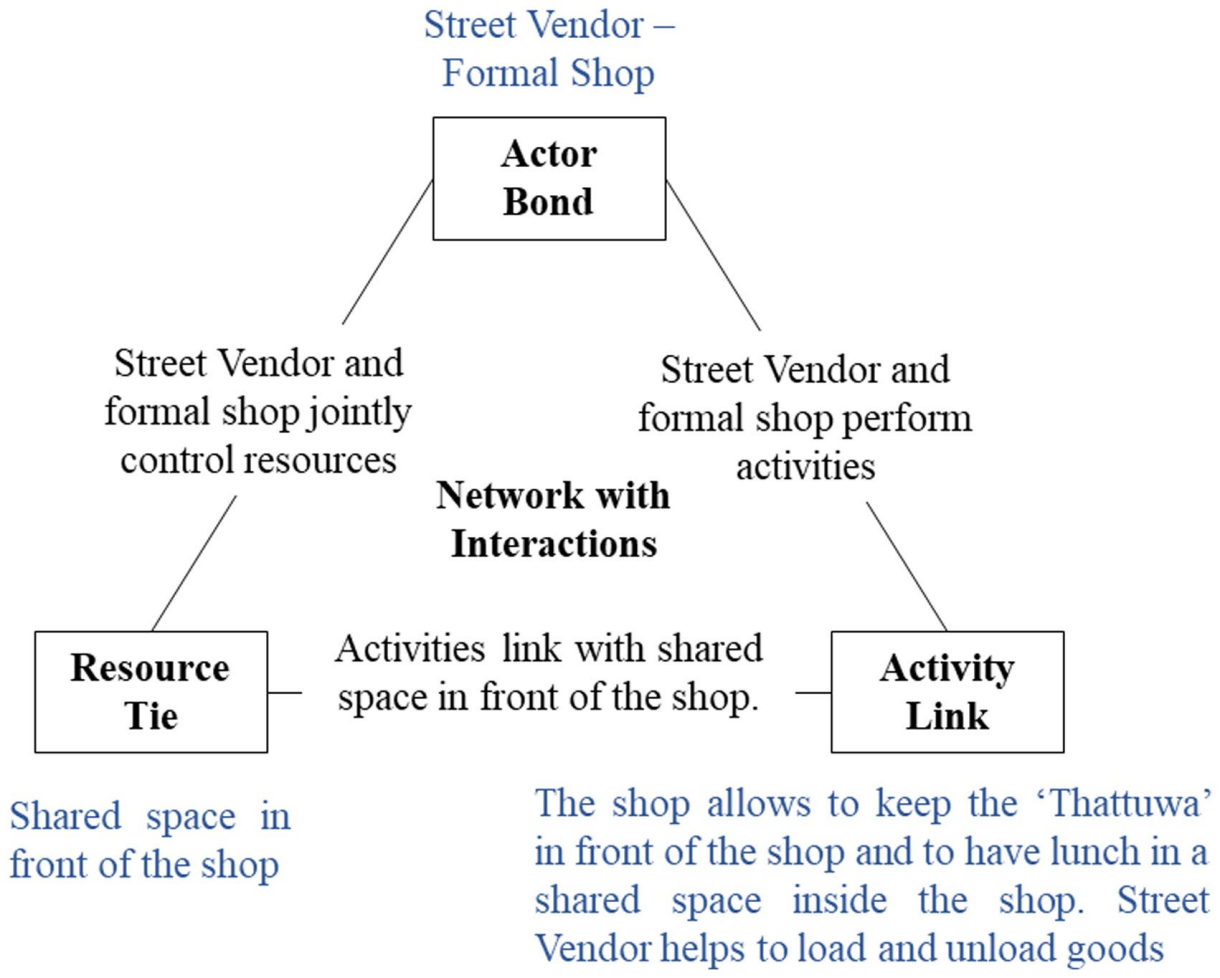
Example 01 with ENM. Source: prepared by the author.

**Table 2:** Identified patterns of the relationship between street vendors and formal urban Space.

No.	Identified pattern of relationship	Example
01	*Direct relationship*	
	A link characterized by direct and uncomplicated interaction between two individuals or entities. This type of relationship can be by prompt and direct contact or engagement, enabling both parties to directly exchange information, resources, or assistance.	Eg: The formal shop (Sandhya Textiles) allows the street vendor (umbrella seller) to keep the ‘Thattuwa’ in front of the shop and to have lunch in a shared space inside the shop (Figure 4).
02	*Indirect relationship*	
	A relationship between two individuals or parties that is established due to another purpose, activity, or interaction. Indirect relationships can enhance an individual’s network reach and provide access to a wide range of resources. However, these ties may include less personal interaction and slower exchange of information when compared to direct relationships.	Eg: The street vendor’s (reed and rushware seller) presence near the school (St. Aloysius College) has created a safer environment for schoolchildren (Figure 5).
03	*Uni-directional relationship*	
	An asymmetrical relationship characterized by one side providing or exerting influence over the other without receiving any reciprocation or feedback. This relationship is defined by a unidirectional transfer of knowledge, resources, or support, where the recipient does not reciprocate directly to the provider.	Eg: The street vendor (reed and rushware seller) engages in vending activities under the shade of the blind wall of the school (St. Aloysius College) (Figure 6).
04	*Bi-directional relationship*	
	A reciprocal relationship (with an interdependent and mutually beneficial nature) is formed when both parties engage in mutual interaction and exert influence on each other. This can be identified as a symbiotic relationship that is formed when individuals or groups rely on each other for specific benefits or support, resulting in mutual advantages for both parties and a reciprocal exchange of information, resources, or support, which enables both parties to derive mutual benefits and actively participate, thus improving the wellbeing, abilities, or social status of both individuals within the network.	Eg: The street vendor (Reed and rushware seller) fulfils the daily needs of the parents until their kids leave school. The presence of parents around the street vendor is advantageous to improve the business (Figure 7).

Source: prepared by the author.

**Table 3:** Identified patterns of the relationship between street vendors and formal urban space under the selected examples.

Ex	Actor bond	Resource tie	Activity link	Nature of the relationship			
				Direct relationship	Indirect relationship	Uni-directional relationship	Bi-directional relationship
01	Street Vendor (Umbrella Seller) -Formal Shop (Sandhya Textiles)	Shared space in front of the shop	The formal shop allows the street vendor to keep the ‘Thattuwa’ in front of the shop and to have lunch in a shared space inside the shop. The street vendor helps to load and unload goods.	✓			✓
02	Street Vendor (Reed and rushware seller)—Parents	Pedestrian path under the shade of the school wall as a shared space	The street vendor fulfils the daily needs of the parents until their kids leave school. The presence of parents around the street vendor is advantageous to improve the business.	✓			✓
	Street Vendor (Reed and rushware seller)—School (St. Aloysius College)	Pedestrian path under the shade of the school wall as a shared space	The street vendor engages in vending activities under the shade of the blind wall of the school.		✓	✓	
	Street Vendor (Reed and rushware seller)–School children	Pedestrian path under the shade of the school wall as a shared space	The street vendor’s presence in that place has created a safer environment for schoolchildren		✓	✓	

Source: prepared by the author.

The second phase of the analysis was conducted quantitatively using SNA. In other words, SNT supplies the interpretive lens applied to the results, while SNA provides the operational toolkit applied to the ENM-coded data from Phase 1. To construct the network dataset for SNA, the actor pairs and ENM layers (actor bond, resource tie, activity link) identified from the activity map in Phase 1 were transformed into a node-and-edge list used to generate the Social Network Diagram (SND). Each unique actor recorded in the coded interviews and observations (e.g., street vendors, formal shop owners, and other urban actors such as schools, pedestrians, or the municipal council) was entered as a ‘Node’. An ‘Edge’ (tie) was created between two nodes wherever at least one ENM layer had been coded between that actor pair in Phase 1, so that the presence of a coded actor bond, resource tie, or activity link was treated as evidence of a network tie for SNA purposes. Each edge was then assigned a weight based on the directionality established during coding: an edge was weighted 2 where the coded relationship was uni-directional, and weighted 4 where it was bi-directional (i.e., where mutual, reciprocal flows were coded in both directions, as summarized in Tables 2, 3). This produced a network dataset of 12 nodes and 22 edges, which was entered into the network analysis software Social Network Visualizer (SocNetV) v3.0.4 to conduct a centrality analysis evaluating the importance of street vendors for the functions of formal urban spaces.

**Figure 10:**
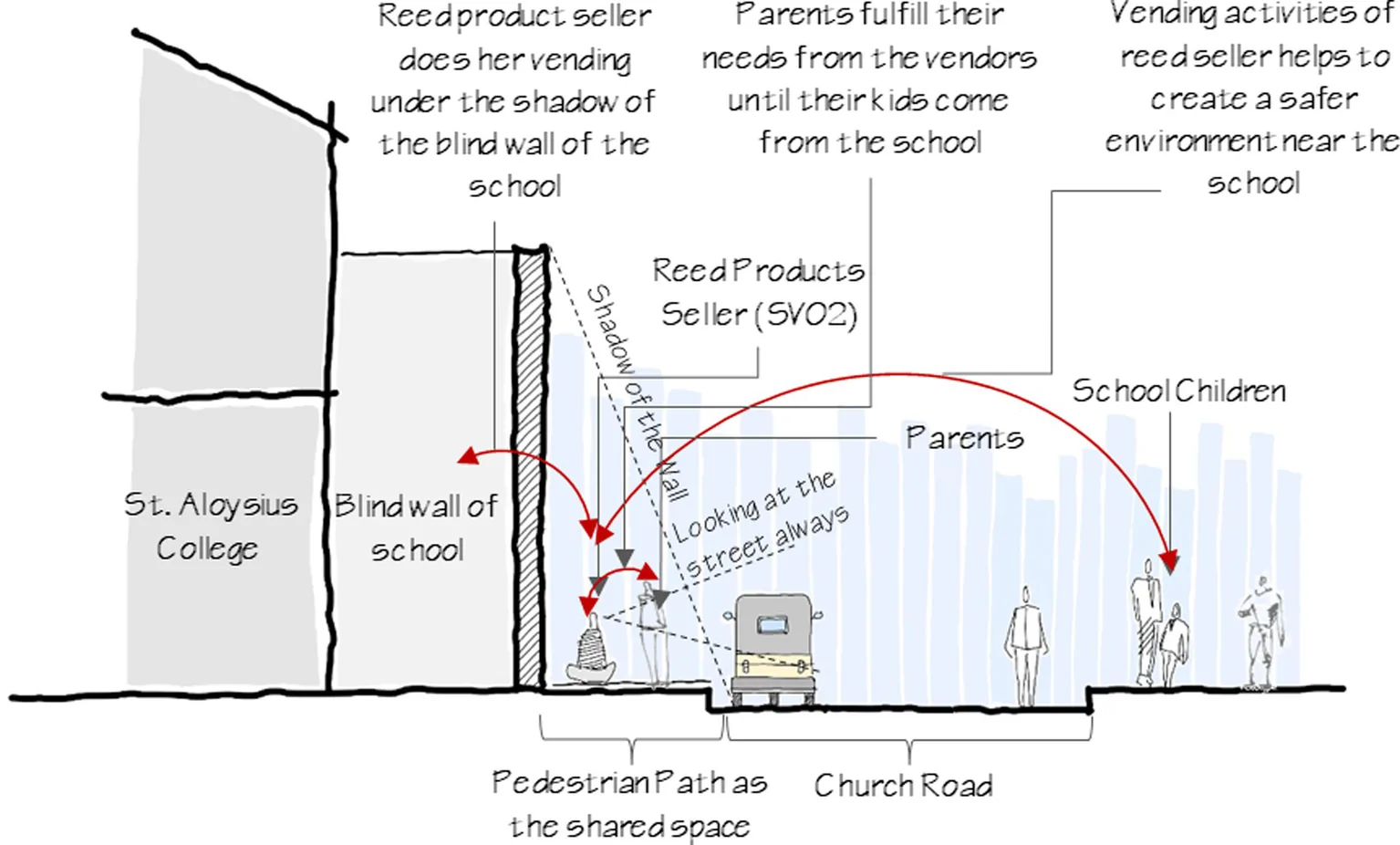
Social network diagram of church road. Source: prepared by the author.

Aligning with this research methodology, the analysis was carried out to understand the relationship between street vendors and formal urban spaces and to explore the invisible networks in the urban economic structure of Church Road.

#### 3.2.1 Ethical disclosure and participant confidentiality

This study was conducted in accordance with ethical research principles concerning the privacy and confidentiality of all participants. Prior to each interview, all 30 participants were informed of the study’s purpose, the voluntary nature of their participation, and their right to decide whether to participate or withdraw at any time. Participation was entirely voluntary, and written consent was obtained from each participant before data collection commenced.

To ensure the complete anonymity of all individuals, no personal identifying information, including names or any other identifiable details, has been recorded with their agreement, and sensitive data such as names, addresses, and contact information were not requested for confidentiality reasons (Nii Laryeafio and Ogbewe, 2023) and were not disclosed or referenced anywhere in this study. Instead, a coding system was employed, wherein street vendors are referred to as ‘SV’ and shop owners/formal vendors as ‘SO’ throughout the analysis. All data collected were used exclusively for academic purposes and are presented in coded or aggregate form to prevent any possibility of individual identification.

## 4 Results

### 4.1 Exploration of the nature of the relationship between street vendors and formal urban space

Based on the collected data, activity mapping is the fundamental technique utilized to comprehend the relationship between street vendors and formal urban space. Subsequently, a comprehensive activity map was generated following the data collected during three distinct periods of the day (Table 1), as shown in Figure 3. Table 1 presents the identified different activities/relationships and the responsible actor for each identified relationship under the relevant time period of the day.

**Figure 11:**
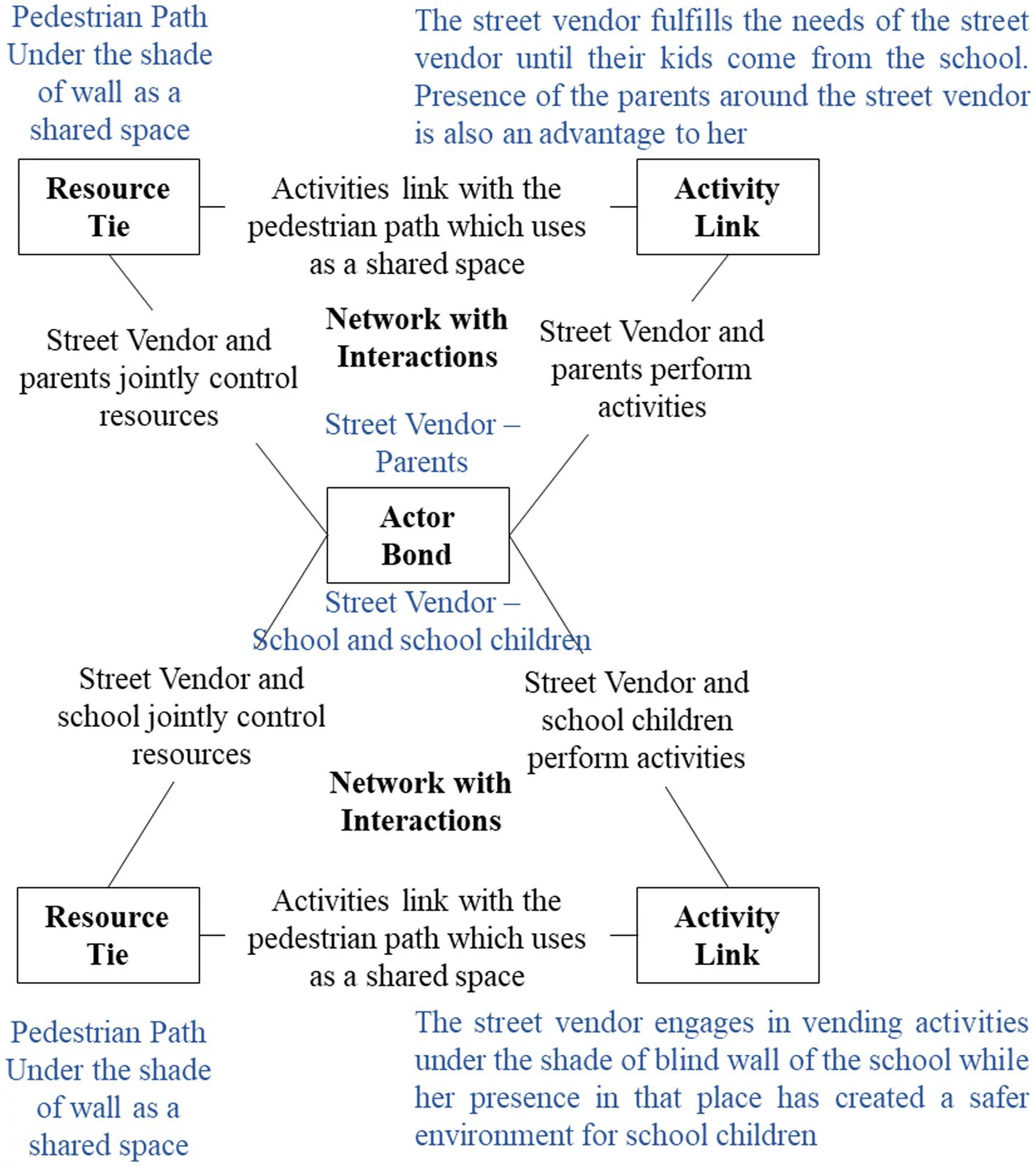
Example 02 with ENM. Source: prepared by the author.

According to the analysis of identified activities and relationships through the activity map, it was revealed that several different patterns of relationships exist in the social network of Church Road based on their nature, as shown in Table 2.

The results of the identified relationships demonstrate that the actors in the social network of Church Road often maintain direct and uncomplicated interactions with each other, particularly the informal street vendors and the actors in the formal urban space. Moreover, it indicates that informal street vendors and formal vendors often share their resources, information, and assistance, as illustrated by the statement: “Yes, they helped us a lot. They allowed me to do my business in front of their shop, and they allowed me to have my meals inside their shop” (Respondent SV04 – Plastic goods seller).

Moreover, the relationships between different actors in the social network of Church Road often create various indirect relationships among them. This means that some relationships among the actors are established for another purpose, activity, or interaction. Evidently, one street vendor stated, “It’s so beneficial to do my vending activities in front of the school because school children are often attracted to buy my pickle” (Respondent SV03 – Pickle seller). Another street vendor remarked, “I used to do business in different places. But now, I’m doing my business in front of the school, because parents who come to pick up their children from the school are often attracted to my place as well” (Respondent SV09 – Watermelon Seller).

Apart from that, this study identifies another relationship pattern involving a uni-directional transfer of knowledge, resources, or support, where the recipient does not reciprocate directly to the provider in the relationship between informal street vendors and formal urban spaces. As a shop owner responded, “we allow the street vendors to keep their goods, and structures inside our shop during the night” (Respondent SO02 – Textile Shop Owner). However, from his response, it is clear that the formal shop owner is supporting the informal street vendor, even though he did not receive any payback or advantage. Moreover, another relationship pattern is identified when individuals or groups rely on each other for specific benefits or support, resulting in mutual advantages for both parties while exchanging information and resources. These types of mutual benefits are clearly visible in the social network of Church Road: “Those shops help me keep the goods. So that’s the kind of help there is, and I often help them to load and unload the goods as well” (Respondent SV11 – Betel Seller); “The seller in that shoe shop helps me to clear this space in the evening and keep my Thattuwa (vending structure) at his shop during the night” (Respondent SV10 – Corn Seller).

As another significant finding of this study, it can be identified that shared spaces between informal street vendors and formal urban actors play an important role in generating these activity links and relationships. These spaces create a platform for easy and consistent interaction, as evidenced by: “They allowed me to do my business in front of their shop and they allow me to have my meals inside their shop” (Respondent SV04 – Plastic Goods Seller) and “It’s so beneficial to do my vending activities in front of the school because school children often come to buy my pickle” (Respondent SV03 – Pickle Seller).

The reasons for these identified patterns were then coded based on three themes: actor bond, resource tie, and activity link, derived from Hakansson’s Economic Network Model. A few selected examples are shown in Table 3.

Accordingly, for the qualitative understanding of the nature of the relationship between formal urban spaces and street vendors, the above thematically coded reasons for the relationship patterns of several exemplary spots of street vending on Church Road are illustrated under Hakansson’s Economic Network Model, as shown below.

#### 4.1.1 Street vendor who sells umbrellas in a shared space in front of a formal shop

The nature of this relationship and interaction between the street vendor and the formal shop is interpreted below based on Hakansson’s Economic Network Model (Figures 8, 9).

The actor bond in the above instance has been established between the formal shop (Sandhya Textiles) and the street vendor (Umbrella Seller – SV06). The resource tie between the actor and the shop is primarily a shared area in front of the shop, where the formal shop has granted the street vendor permission to store his ‘Thattuwa’ and conduct his vending activities. In addition to this, the formal shop has allocated a communal area within itself for the street vendor to consume his meals. Furthermore, the street vendor assists in the formal store during the unloading of goods from the trucks. The resource ties between these actors generate activity links in the shared realm.

The activity links of this relationship imply that they are generated in both directions, and both actors gain direct benefits from this relationship. Consequently, based on these three ENM-demonstrated layers (actor bonds, resource ties, activity links), a mutually beneficial and interdependent relationship, along with direct interaction, exists between the street vendor and the formal store.

#### 4.1.2 A reed and rushware seller engages in her vending activities under the shade provided by the wall of a nearby school

In this relationship, there are several actor bonds that have fostered interactions such as street vendor-school, school children, and street vendor-parents. Accordingly, the resource tie where the activity links are generated in these relationships is the pedestrian path as a shared resource. The street vendor engages in her vending activities under the shade of the blind wall of St. Aloysius College, which is a relationship that occurs only in one direction. Her presence in that location for vending activities is important and mutually beneficial for both actor bonds. Consequently, the street vendor near the school has created a safer and livelier environment through her activities. Furthermore, through that activity link, the parents who come to pick up their children fulfil their needs while waiting for the children to come from school, indicating a direct relationship in both directions. Thus, it can be interpreted that this street vendor is, directly and indirectly, interconnected and mutually interdependent with the actors in formal urban space in various ways (Figures 10, 11).

**Figure 12:**
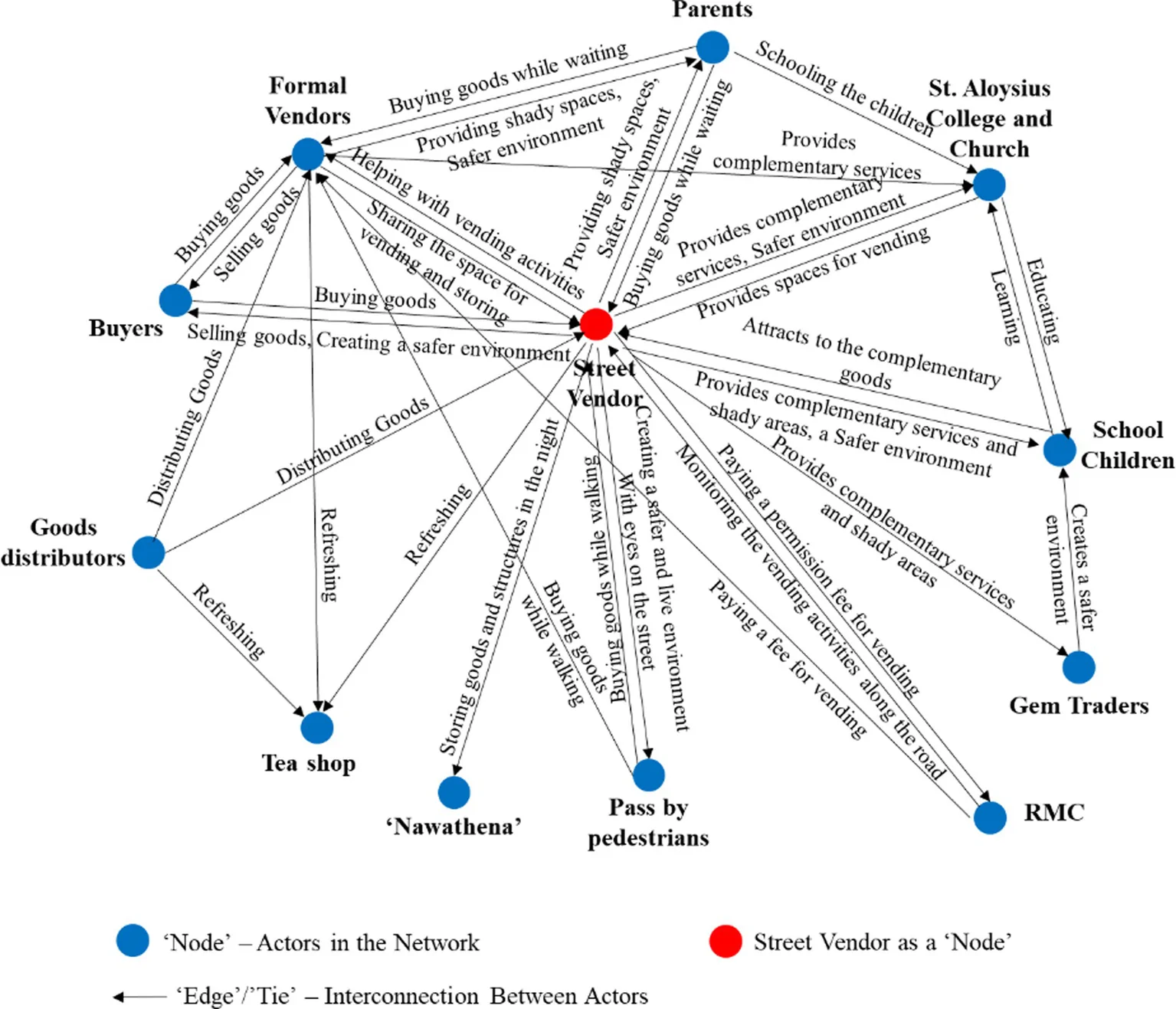
Social Network Diagram of Church Road. Source: prepared by the author.

**Figure 13:**
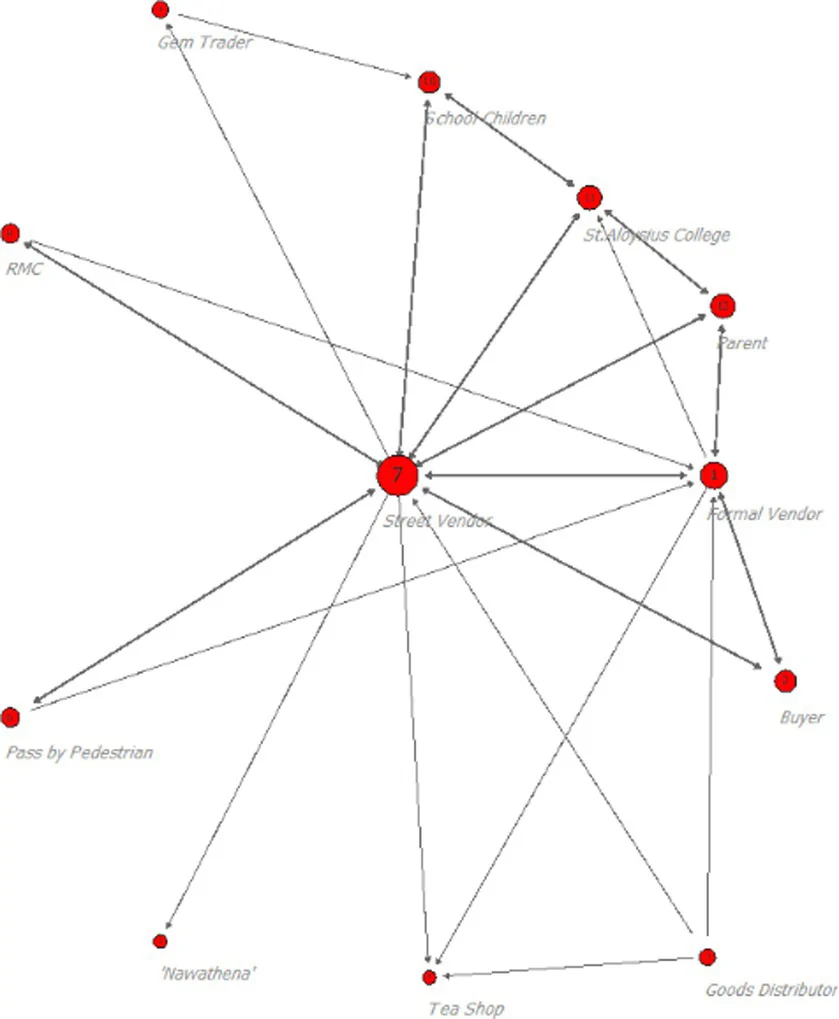
Degree Centrality. Source: prepared by the author.

With all this, it can be identified that the street vendors and formal urban spaces are interconnected, and the nature of their relationships is based on various mutual interdependencies. Street vendors are an inseparable urban element of the formal urban space and the formal urban economic structures of Church Road.

Moreover, activity mapping and ENM analysis indicate that the social network of Church Road is complex, with street vendors occupying a critical position by establishing relationships with the majority of other actors in formal urban environments. This relationship between street vendors and the formal urban environment is marked by mutual dependence and reciprocity, illustrating that the operation and essence of Church Road’s formal spaces are deeply intertwined with the activities of street vendors.

### 4.2 Evaluation of the importance of street vendors for the functions of formal urban spaces

Based on the results of qualitative analysis, various actors and relationships were identified. During Phase 2 of the analysis, the identified actors are considered as ‘Nodes’ while relationships are considered as ‘Edges/Ties’, and the Social Network Diagram (Figure 12) was prepared to illustrate the social network of Church Road. Accordingly, this section presents the analysis and results of network and centrality analysis. First, the general properties of the graph are discussed, and three centrality measures are presented. In each case, the values for the nodes are provided in a table. Finally, the results of the centrality measures are compared and discussed with regard to the spatial context of the studied social network of Church Road.

**Table 4:** Results of the centrality analysis.

Node/actor	Degree centrality	Betweenness centrality	Closeness centrality
	DC value	BC value	CC value
Formal vendor	14.000000	5.000000	15.000000
Buyer	8.000000	0.000000	8.000000
Goods distributor	3.000000	0.000000	0.000000
Tea shop	0.000000	0.000000	3.000000
‘Nawathena’	0.000000	0.000000	1.000000
Pass by pedestrian	5.000000	0.000000	4.000000
Street vendor	31.000000	62.500000	29.000000
RMC	5.000000	0.000000	4.000000
Gem trader	1.000000	0.000000	1.000000
School children	8.000000	9.000000	9.000000
St. Aloysius college	12.000000	1.000000	13.000000
Parent	12.000000	0.000000	12.000000

Source: generated by the author using SocNetV (v3.0.4).

#### 4.2.1 Network and graph results

The network constructed in this study consists of 12 nodes and 22 edges, and the basic parameters of the network are as follows: each node represents the actors in the social network of Church Road, and the edges represent the connections between actors. The edges are weighted as 2 when there is only one interconnection (uni-directional relationship) between two nodes, while the edge is weighted as 4 when there is more than one interconnection (bi-directional relationship). The edge weight is particularly useful for interpreting degree centrality (DC), which is based simply on the number of links (Dudzic-Gyurkovich, 2023). The geometry of the graph remains consistent in each case, and the Node Size by Prominence Index determines the layout of the graphs. The results of each centrality calculation are presented in the graphs (Figures 13–15).

**Figure 14:**
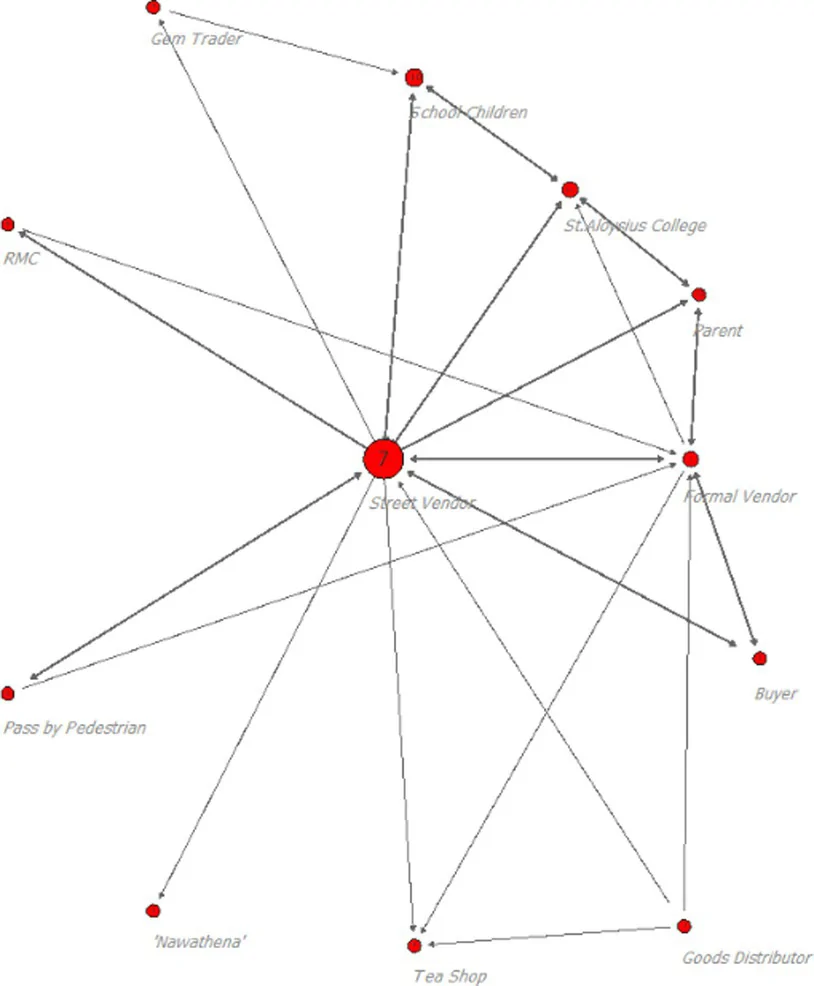
Betweeness Centrality. Source: generated by the author using SocNetV (v3.0.4).

**Figure 15:**
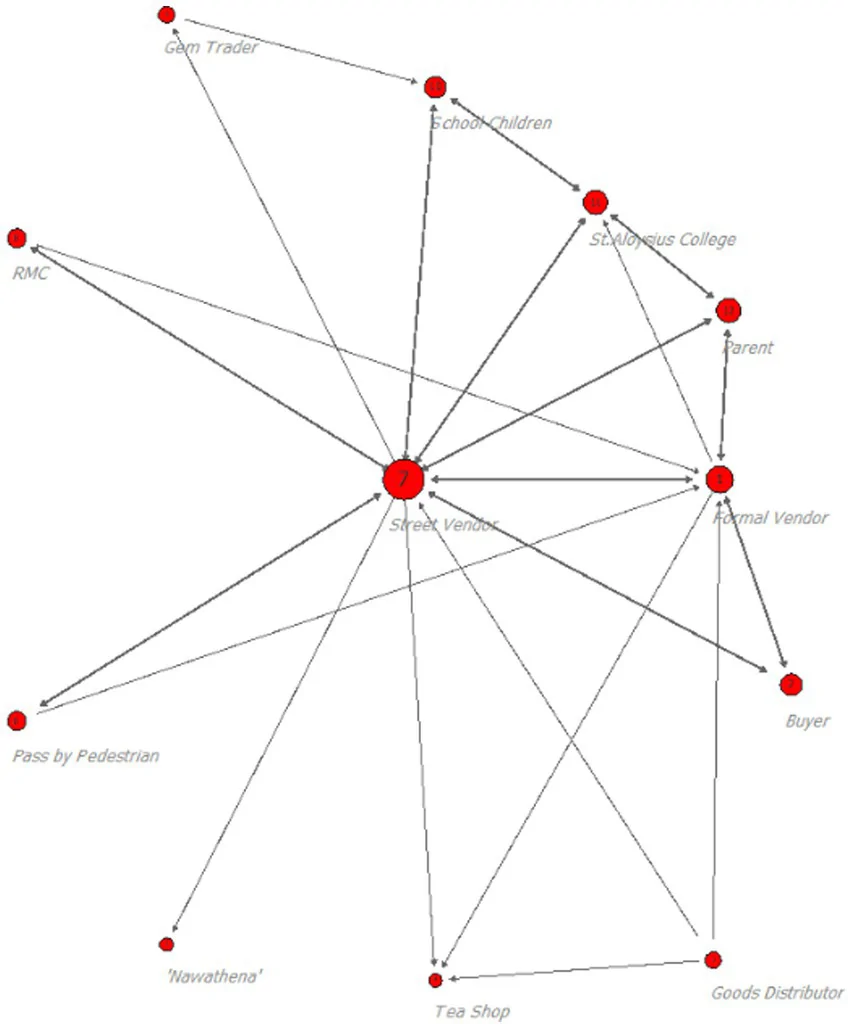
Closeness Centrality Source: generated by the author using SocNetV (v3.0.4).

The results of the degree centrality (DC) range from 0 for the unconnected nodes to over 31 for the most connected nodes (Table 4).

The highest value (31) in this case is assigned to the node of Street Vendors, followed by 14 for the node of the formal vendor and 12 for the nodes of St. Aloysius College and Parents (Figure 13, Table 4). The results indicate that these nodes have the largest number of potential connections within their social network, and their high level of connectivity underscores their critical role in facilitating interactions and economic transactions, especially the Street Vendors, the node with the highest DC value. The numerous connections that street vendors maintain with other actors, such as buyers, formal vendors, and pedestrians, highlight their essential function in sustaining the flow of economic activities and social interactions. For instance, street vendors on Church Road directly connect with Good Distributors, stating, “I get the vegetables to sell from a distributor on a daily basis” (Respondent SV21 - Vegetable Seller), and with Formal Vendors, who note, “they allowed me to do my business in front of their shop and they allow me to have my meals inside their shop” (Respondent SV04 - Plastic Goods Seller). Additionally, it was observed that these street vendors often interact with the well-known Tea Shop at Church Road, ‘Nawathena’, where most of the vendors store their goods, as well as with gem traders and the Ratnapura Municipal Council. This prominence implies that street vendors have a substantial impact on the circulation of products and the vitality of the local economy, as revealed during the qualitative interviews: “the ones who are coming back from their work in the evening often fulfil their daily needs from us while they are going to the bus stand” (Respondent SV08 - Fruits Seller). Accordingly, it is clear that street vendors are a central node within the social network of Church Road, boasting the highest number of direct connections with other actors in the network. The presence of street vendors attracts significant foot traffic, supports formal businesses by increasing customer visits, and contributes to the overall vibrancy and dynamism of the urban environment.

The next measure was the betweenness centrality (BC), which ranges from 0 to 62.5 (Figure 14, Table 4). In this case, the results showed significant differences. There is only one node with the highest score of 62.5 (Street Vendors), while there are also eight nodes that received a score of zero, meaning that they were never on the path connecting other nodes and are positioned on the edges of the network. These results suggest that nodes with the highest scores can act as ‘bridges’ between other nodes, facilitating the flow of goods, information, and interactions among various urban actors. By serving as intermediaries, street vendors bridge connections between otherwise disconnected parts of the network, ensuring efficient resource distribution and communication. Their intermediary role emphasizes their significant influence in maintaining the functionality and resilience of the urban economic system. For instance, “People come here to buy goods because they have the impression that goods are much more affordable here due to the street vendors, so we also gain that benefit of attracting more customers to our shops” (Respondent SO08 – Textile Shop Owner) and “I think it’s an advantage. Because people get the impression that there are a lot of goods, then many people come to this site to buy goods” (Respondent SO04 – Textile Shop Owner), implying that street vendors act as intermediaries inthe relationship between formal shops and buyers/customers. Furthermore, one of the significant findings of this study is that the indirect nature of the relationships between informal street vendors and formal urban actors occurs especially when the same shared space is used by both actors, creating a platform for informal street vendors to bridge the relationship between different formal urban actors. Evidently, “I used to do business in different places. But now, I’m doing my business in front of the school because parents who come to pick their children up from the school are often attracted to my place as well” (Respondent SV09 – Watermelon Seller).

The closeness centrality (CC) results show 0 as the lowest value and 29 as the highest (Figure 15, Table 4). The CC values show variability, with only four nodes reaching the highest value possible, while the majority of nodes range between 0 and 10. The highest value (29) was achieved by the node of Street Vendors, the next value of 15 was assigned to the node of Formal Vendor, and finally, 13 and 12 were assigned to the nodes of St. Aloysius College and parents, respectively. Street vendors, with the highest value of CC, are positioned as highly influential nodes within the network. This proximity allows street vendors to rapidly engage with and influence other actors, facilitating swift interactions and transactions. Their strategic location enables them to act as hubs of activity, promoting essential economic and social exchanges that contribute to the vibrancy and functionality of formal urban spaces. The ability of street vendors to efficiently interact with multiple actors makes them indispensable for the dynamic functioning of the urban economy.

## 5 Discussion

Through the application of Hakansson’s Economic Network Model (ENM) and centrality analysis under Social Network Theory (SNT), this study identified different types of relationships and interactions between informal street vendors and formal urban spaces, and provided insight into how these informal street vendors are important to the continuity of formal urban spaces. The findings provide a basis for understanding the role within the social network of Church Road, and, taken together with recent literature, point to a broader methodological and theoretical contribution beyond this specific empirical setting.

### 5.1 Nature of relationship patterns between informal street vendors and formal urban spaces

The findings of the application of Hakansson’s Economic Network Model identified four different types of relationships: (1) direct relationship, (2) indirect relationship, (3) uni-directional Relationship, and (4) bi-directional relationship. These four types of relationships were derived based on the way they share resources and the flow of information, considering the actor bond, resource tie, and activity link in Hakansson’s Economic Network Model.

This finding agrees with a study conducted on patterns of power relations of street vendors in Bandung City, which similarly emphasized how street vendors maintain symbiotic relationships with other actors in their network (Hermawati and Paskarina, 2020), and is consistent with a recent inter-scale study of informal street vending networks in the Peruvian Amazon, which likewise found that street vendors are embedded in dense webs of commercial and social ties connecting urban and territorial scales, rather than operating as isolated economic units (Mendoza-Santa Cruz et al., 2025). However, this finding contradicts the study that demonstrated the contestation of urban public spaces due to the activities of street vendors in Sri Lanka (Herath, 2015), suggesting that the balance between cooperative and contested relations may vary considerably even within the same national context, depending on the specific urban setting and scale studied.

As found, there are no significant studies on the identification of indirect relationships between informal street vendors and formal urban spaces. Similarly, no study to date has examined the uni-directional and bi-directional relationships between informal street vendors and formal urban spaces; these two relationship types were previously identified by Hernandez and Johnston (2016) in a study of children’s behavior, but not from the perspective of the spatial context of different actors in a social network, nor in relation to informal economic activity. This gap has persisted even as recent research on street vendor entrepreneurship has begun to recognize networking and relationship-building as core informal economic skills (Awapak et al., 2025), without yet disaggregating those relationships into the specific structural types identified in this study. Noticeably, there are also no specific studies conducted to understand the contribution of shared spaces to generating these relationship types, despite recent geo-spatial research confirming that informal vendor clustering is itself strongly shaped by shared and contested urban space (Hu et al., 2026).

Considering all this, the present study clearly emphasizes the various relationships generated among the different urban actors and informal street vendors. These findings pave the way for understanding the importance of informal street vendors and their role in the social network of Church Road, which is discussed in the following section.

### 5.2 Importance of informal street vendors in the social network of church road

This research highlights the profound impact of street vendors on urban economies and social structures, drawing contrasts and making new connections with existing literature.

As the actors with the highest degree centrality, street vendors were found to have the most direct connections or relationships with other actors in the social network of Church Road. This finding is reinforced by the relationship patterns discussed above. Compared to the scholarly literature, most studies about street vendors have focused on direct connections solely through the lens of economic or business aspects rather than the spatial aspect (Karunaratna and Tjandra, 2021). This study addresses that gap by situating direct economic ties within their spatial and relational context.

Moreover, this study reveals that street vendors serve as intermediaries within the social network of Church Road, as evidenced by their high betweenness centrality. This indicates that street vendors have a comparatively greater capability to influence the functions of Church Road. Nayomi and Mahanama (2014) similarly found that informal social groups are influential for the functioning of formal urban spaces, in a study of Natamis in Pettah, Sri Lanka. This result regarding informal street vendors is supported by the indirect relationships identified through Hakansson’s Economic Network Model, as street vendors often act as brokers, bridges, or intermediaries between the activity links of various urban actors, facilitating indirect benefits, services, or functions. This intermediary role reflects recent findings from Southern Thailand, where street vendor groups were similarly found to strengthen local economic circulation by connecting formal institutions, financial actors, and informal traders through a shared network structure (Awapak et al., 2025). This reinforces that the brokering function identified in Church Road is not an isolated case but part of a broader pattern observable across other Global South street vending economies.

Furthermore, the high closeness centrality of informal street vendors emphasizes that they are the closest nodes or actors to all other actors in the social network of Church Road, along the shortest path of the network. This identifies street vendors as critical nodes contributing to the network’s overall efficiency. According to the scholarly literature, several studies have examined closeness centrality in street networks (Crucitti et al., 2005; Rui and Ban, 2014; Liu et al., 2015), in different urban networks (Agryzkov et al., 2019), and in pedestrian movements (Pont et al., 2019). However, no significant study to date has measured the closeness centrality of street vendors specifically within a social network, positioning this finding as an original contribution of the present study.

An important finding of this research is that the relationships between street vendors, formal vendors, and city users extend beyond economic exchanges and are strongly rooted in social capital. It is well established that street vendors play an important role in the urban economy, particularly in the informal economy (Bhowmik and Saha, 2012; Roever and Skinner, 2016; Martínez and Short, 2022). However, only a few studies have examined the social capital of street vendors (Lyons and Snoxell, 2005). This research extends the existing body of knowledge by not only identifying the social capital of street vendors but also analysing the patterns of their social networks and the underlying economic structures.

This study further indicates that the social relationships of street vendors are influenced by spatial configuration. Previous studies have shown that space provides the basis for social relations and that spatial configuration plays a central role in creating opportunities for social interaction (Tonkiss, 2005; Small and Adler, 2019; Qi et al., 2024; Meetiyagoda et al., 2024). Although research on street vending has explored the spatial logic of informal street vending (Peimani and Kamalipour, 2022; Rahayu et al., 2025), it has only limitedly examined the relationship between the spatial positions of street vendors and their social networks.

## 6 Conclusion

This study aimed to explain the functions of street vendors in the urban economic network of Church Road, Ratnapura, and was the first to apply Hakansson’s Economic Network Model (ENM) in the context of informal street vendors, incorporating Social Network Analysis (SNA) as per the referred literature. The main findings of this research are: (1) four different types of relationships: direct relationship, indirect relationship, uni-directional relationship, and bi-directional relationship. Furthermore, the findings of this study elaborate that, most of the time, the relationships amongdifferent actors in the social network of Church Road create various interactions among them. This means that some relationships among the actors are established for another purpose, activity, or interaction. Most frequently, the shared spaces are the major reason for the occurrence of these relationships. Additionally, the findings are strengthened by revealing how street vendors occupy a central role within the social network of Church Road, with the highest number of direct connections or relationships with other actors (DC value), and street vendors function as intermediaries within the social network of Church Road, as evidenced by the highest value of betweenness centrality. Furthermore, informal street vendors, showing the highest closeness centrality, emphasize that they are the closest nodes or actors to other members of the social network of Church Road, along the shortest path of the network.

The findings also contribute to the literature on informal–formal relationships by demonstrating that these relationships are characterized not only by competition but also by cooperation, interdependence, and mutual value creation. Street vendors function as intermediaries, connecting multiple actors through actor bonds, activity links, and resource ties, highlighting the embedded nature of informal economic activities within the formal urban economy.

Methodologically, the integration of Håkansson’s Economic Network Model with Social Network Analysis provides a novel framework for examining informal economies. While Social Network Analysis identifies the structural positions of actors within the network, the Economic Network Model explains the underlying actor bonds, activity links, and resource ties that generate these network structures. Together, these approaches reveal the complexity of socio-economic interactions that cannot be understood through purely spatial or economic analyses.

Understanding these varied relationship patterns and the importance of conserving their shared spaces is crucial for urban planners and policymakers in various urban interventions. Moreover, these insights illustrate the interconnectedness and dependencies within the urban economic fabric, which can inform policies that foster inclusive urban development. By recognizing and supporting these relationships, planners can ensure that both formal and informal economic activities are integrated and mutually supportive. Meanwhile, the findings from Ratnapura may have broader relevance for urban areas with similar socio-economic and spatial characteristics across the Global South. Like many secondary cities, Ratnapura exhibits recurring daily activity patterns, including morning commuting, lunchtime peaks, afternoon lulls, evening congestion, and nighttime wind-downs, which shape the temporal dynamics of street vending. In addition, vending activities are concentrated around major activity nodes such as transport hubs, hospitals, schools, and commercial markets, where high levels of pedestrian movement support informal trading. Therefore, while the specific network structures identified in Ratnapura may not be directly transferable to all contexts, the underlying socio-economic relationships between street vendors, formal vendors, and urban users are likely to be relevant in cities with comparable spatial and economic conditions.

Although the study provides valuable insights, it is important to acknowledge its limitations. The confined dependence on one specific geographic area and the comparatively limited number of participants may not fully encompass the wide range of street vending dynamics. Moreover, the subjective nature of the data could potentially lead to biased interpretations of the social and economic impacts of street vendors. Subsequent studies should investigate various urban contexts to validate the applicability of these findings. Further quantitative studies could also provide a broader statistical foundation to support the development of targeted urban policies. Furthermore, researching the influence of advances in technology and digital platforms on the street vending industry could provide a more profound understanding of forthcoming urban economic models.

Finally, the findings suggest that street vendors should be recognized as legitimate stakeholders in urban governance rather than merely as unauthorized users of public space. Since they occupy central positions within urban economic networks, interventions such as relocation or eviction may have cascading effects on formal businesses, customers, and suppliers. Urban governance should therefore adopt participatory approaches that incorporate street vendors into decision-making processes concerning the management and regulation of public spaces.

## Data Availability

The original contributions presented in the study are included in the article/supplementary material, further inquiries can be directed to the corresponding author.
